# Quality and accessibility of online patient self-education resources for breast reconstruction

**DOI:** 10.1016/j.jpra.2025.12.022

**Published:** 2025-12-18

**Authors:** Arashk Ghasroddashti, Colm Guyn, Yonatan Fortinsky, Robert Wesley Edmunds, Glykeria Martou

**Affiliations:** a School of Medicine, Queen’s University, Kingston, ON, Canada; b Faculty of Medicine and Dentistry, University of Alberta, Edmonton, AB, Canada; c Faculty of Medicine, University of Ottawa, Ottawa, ON, Canada; d Division of Plastic Surgery, Department of Surgery, Queen’s University, Kingston, ON, Canada

**Keywords:** Patient education, Breast reconstruction, Health information, Internet, Google

## Abstract

**Background:**

With rising interest in breast reconstruction after mastectomy, patients are increasingly turning to online resources to supplement medical consultations. However, the quality and accessibility of these materials remain inconsistent. This study evaluates the readability, understandability, actionability, content coverage, and transparency of online breast reconstruction resources.

**Methods:**

The top 20 Google search results were examined for five common breast reconstruction-related queries. Metrics assessed included SMOG readability level, PEMAT scores (understandability and actionability), content coverage, and a modified EQIP score for quality. Statistical analyses examined relationships among these variables and with factors like search rank, author type, and query.

**Results:**

Mean content coverage was 49 %, with significant gaps in preoperative planning, treatment side effects, and fat grafting. Readability was poor (mean SMOG 12.3). Understandability was high (80 %), but actionability (37 %) and quality (modEQIP of 40 %) were low. Academic authors produced shorter and lower-quality resources. Higher-ranked resources were generally longer and correlated with better performance across most metrics. Specific queries like ‘DIEP flap’ yielded narrower, lower-quality resources.

**Conclusions:**

Online resources for breast reconstruction are highly variable and often fall short in readability, comprehensiveness, and transparency. Although understandability is generally acceptable, low actionability and inconsistent coverage hinder patient utility. Search engine rank modestly correlates with quality, suggesting some alignment between visibility and value. Improving these resources will require targeted efforts to simplify language, address topic gaps, and enhance actionable content—especially for specialized queries where quality remains lowest.

## Introduction

Breast cancer remains one of the most common malignancies in women.^1^ As surgical management and reconstructive techniques have evolved, patient demand for post-mastectomy breast reconstruction has grown—driven in part by increased awareness and access to reconstructive options.^2^

The internet is a major information source for patients considering breast reconstruction, with an estimated 95 % of cancer patients using it to learn about diagnosis and treatment.^3^^,^^4^ Many find that medical visits alone do not address emotional, physical, or practical needs, making online resources an important extension of support.^3^ Such materials allow patients to revisit complex information, explore topics at their own pace, and seek peer guidance, supporting shared decision-making and informed consent within patient-centered care.^5^

Given the potential of online resources to support patients, it is crucial that they provide accurate and understandable information in a user-friendly format. However, the quality of such resources varies considerably in accuracy, readability, and utility.^6^ As such, this study evaluates the overall quality and accessibility of online, text-based breast reconstruction resources using a combination of validated metrics to assess readability, understandability, actionability, content coverage, and quality.^7^, ^8^, ^9^ Previous studies have evaluated some of these metrics individually, often finding they fall below accepted standards.^10^, ^11^, ^12^, ^13^ However, rapid web content turnover and the lack of a comprehensive, relational assessment represent gaps in the existing literature. Examining the aforementioned metrics in relation to one another and to resource characteristics may reveal patterns and guide improvements in online patient education.

## Methods

### Search strategy

Based on search engine market share and breast cancer patient information-seeking practices, Google was used to search the five most common breast reconstruction–related queries, identified via Google Trends.^14^^,^^15^ For each query, the top 20 non-sponsored, English-language results were examined. To minimize variability, all searches were conducted on May 12, 2024 using the Google Chrome browser Incognito mode, cleared of cache and cookies, with location standardized to the United States. Searches were performed in Kingston, Ontario, Canada.

### Data collection

Two independent reviewers screened all search results; subjective variation was minimized by use of standardized scoring tools. For results with multiple related pages, content was aggregated under a single entry when appropriate. Results within the same subdirectory were treated as duplicates and evaluated only once, except in analyses involving query or rank. Each result’s originating query and search rank were recorded, along with metadata on country of origin, author type, and target audience as categorized in Table 1. Readability features were extracted using an online tool (readabilityformulas.com), which provided the number of words, syllables, polysyllabic words, and sentences per result. These data were then used to calculate readability metrics (see ‘Assessment Tools’). Each result was reviewed for coverage of key breast reconstruction subtopics identified through literature and author consensus (Table 2).^2^^,^^16^ Subtopics received a binary score and an overall content score was calculated as the percentage of subtopics discussed. Readability, understandability, actionability, and quality were then assessed using validated tools (described below).

**Table 1:** Options for resource authorship, target audience, publication type, and country.

Author type	News organization Encyclopedia Government, charitable organization Corporation, professional society Healthcare portal Academic institution Private practice Nonmedical professional Non-MD medical professional Plastic surgeon MD Other MD BR patient or close support Other (layperson)
Target audience	Patients, health professionals Other professionals Health industry Other
Country	United States Canada United Kingdom Australia

**Table 2:** Content topics and frequency of discussion across resources.

Content subtopics	Count of topic discussion across resources (*N* = 47)
Autologous (general)	45
Implant	42
Aesthetic outcome	38
Autologous (DIEP)	38
Complications	30
Autologous (TRAM)	30
Neoadjuvant/adjuvant	28
Nipple areolar complex reconstruction	26
Tissue expansion	26
Post-operative recovery	24
Symmetrizing procedures (excluding fat grafting)	16
Neoadjuvant/adjuvant adverse effects	14
Recurrence	14
Pre-operative preparation	14
Fat grafting	12
Meshing/acellular dermal matrix	12

Abbreviations: TRAM, transverse rectus abdominis myocutaneous, DIEP, deep inferior epigastric perforator artery.

### Assessment tools

Readability was assessed using the Simple Measure of Gobbledygook (SMOG) formula, which estimates the U.S. grade level required to comprehend a text using the formula :$1.043\times \sqrt{30\times \frac{Polysyllabic\;Words}{Sentence}+3.1291}$.^8^ The Patient Education Materials Assessment Tool for printable materials (PEMAT), was used to evaluate understandability (PEMAT-U, items 1–17) and actionability (PEMAT-A, items 18–24) (Table 3).^7^ Understandability reflects clarity of content, language, structure and visual aids, while actionability assesses whether materials provide clear, specific instructions.^7^ Items were scored 0 (disagree), 1 (agree), or N/A, and percentage scores were calculated for PEMAT-U, PEMAT-A, and overall PEMAT. Quality was assessed using a modified version of the Ensuring Quality Information for Patients (modEQIP) instrument, based on items 18–24, which evaluate authorship, sourcing, and transparency (Table 4).^9^ Items were rated 0 (no), ½ (partly), 1 (yes), or N/A and a percentage score was calculated. All assessments were conducted by two independent reviewers.

**Table 3:** Patient Education Materials Assessment Tool (PEMAT) for printable materials.

Item #	Topic	Question	Response options
1	Content	The material makes its purpose completely evident.	Disagree = 0, Agree = 1
2	Content	The material does not include information or content that distracts from its purpose.	Disagree = 0, Agree = 1
3	Word choice & style	The material uses common, everyday language.	Disagree = 0, Agree = 1
4	Word choice & style	Medical terms are used only to familiarize the audience with the terms. When used, medical terms are defined.	Disagree = 0, Agree = 1
5	Word choice & style	The material uses the active voice.	Disagree = 0, Agree = 1
6	Use of numbers	Numbers appearing in the material are clear and easy to understand.	Disagree = 0, Agree = 1, No numbers = N/A
7	Use of numbers	The material does not expect the user to perform calculations.	Disagree = 0, Agree = 1
8	Organization	The material breaks or "chunks" information into short sections.	Disagree = 0, Agree = 1, Very short material = N/A
9	Organization	The material's sections have informative headers.	Disagree = 0, Agree = 1, Very short material = N/A
10	Organization	The material presents information in a logical sequence.	Disagree = 0, Agree = 1
11	Organization	The material provides a summary.	Disagree = 0, Agree = 1, Very short material = N/A
12	Layout & design	The material uses visual cues (e.g., arrows, boxes, bullets, bold, larger font, highlighting) to draw attention to key points.	Disagree = 0, Agree = 1, Video = N/A
13	Use of visual aids	The material uses visual aids whenever they could make content more easily understood (e.g., illustration of healthy portion size).	Disagree = 0, Agree = 1
14	Use of visual aids	The material’s visual aids reinforce rather than distract from the content.	Disagree = 0, Agree = 1, No visual aids = N/A
15	Use of visual aids	The material’s visual aids have clear titles or captions.	Disagree = 0, Agree = 1, No visual aids = N/A
16	Use of visual aids	The material uses illustrations and photographs that are clear and uncluttered.	Disagree = 0, Agree = 1, No visual aids = N/A
17	Use of visual aids	The material uses simple tables with short and clear row and column headings.	Disagree = 0, Agree = 1, No tables = N/A
18	Actionability	The material clearly identifies at least one action the user can take.	Disagree = 0, Agree = 1
19	Actionability	The material addresses the user directly when describing actions.	Disagree = 0, Agree = 1
20	Actionability	The material breaks down any action into manageable, explicit steps.	Disagree = 0, Agree = 1
21	Actionability	The material provides a tangible tool (e.g., menu planners, checklists) whenever it could help the user take action.	Disagree = 0, Agree = 1
22	Actionability	The material provides simple instructions or examples of how to perform calculations.	Disagree = 0, Agree = 1, No calculations = N/A
23	Actionability	The material explains how to use the charts, graphs, tables, or diagrams to take actions.	Disagree = 0, Agree = 1, No charts, graphs, tables, or diagrams = N/A
24	Actionability	The material uses visual aids whenever they could make it easier to act on the instructions.	Disagree = 0, Agree = 1

**Table 4:** Questions used in the modified ensuring quality information for patients instrument (modEQIP).

EQIP question #	Question	Response options
18	Covers all relevant issues on topic	Disagree = 0, Partly = 0.5, Agree = 1, N/A
19	Contains date of issue or revision	Disagree = 0, Partly = 0.5, Agree = 1, N/A
20	Contains logo of issuing body	Disagree = 0, Partly = 0.5, Agree = 1, N/A
21	Contains name of persons or entities that produced it	Disagree = 0, Partly = 0.5, Agree = 1, N/A
22	Contains name of persons or entities that financed it	Disagree = 0, Partly = 0.5, Agree = 1, N/A
23	Contains short bibliography of evidence-based data used	Disagree = 0, Partly = 0.5, Agree = 1, N/A
24	States if and how patients were involved/consulted in its production	Disagree = 0, Partly = 0.5, Agree = 1, N/A

### Statistical analysis

Descriptive statistics were calculated for year of publication, article length, content coverage, SMOG, PEMAT-U, PEMAT-A, PEMAT, and modEQIP. These statistics were compared across subgroups stratified by query, rank, author type, target audience, and country of origin. Duplicate results were retained in analyses involving query and rank variables.

To analyze the relationships among variables, parametric and non-parametric tests were used. Relationships between ordinal/continuous variables were evaluated using Spearman’s rank correlation coefficient (*ρ*). Categorical and continuous variables were evaluated using a Kruskal Wallis test. Dichotomous and continuous/ordinal variables were evaluated using a Mann-Whitney test. All statistical analyses were performed using SPSS Statistics version 29.0.1.0 (IBM Corp, Armonk, NY). A *p*-value of 0.05 was taken to indicate significance.

## Results

'Breast reconstruction' was used as the primary search term on Google Trends to identify the four other most common queries related thereto. These were: ‘DIEP flap,’ ‘mastectomy reconstruction,’ ‘breast reconstruction surgery,’ and ‘mastectomy breast reconstruction.’ Taking the 20 top results from each query, 47 unique resources were identified. No major discrepancies arose during evaluation, and any minor discrepancies were addressed by discussion and consensus.

Resources were published or updated between 2016 and 2024 and originated in the United States, Canada, Australia, or the United Kingdom. Twenty resources had academic authors. Patients were the target audience in 43 resources, while healthcare providers were the target audience in three cases.

### Content

Among unique resources, content coverage was 49 % ± 25 % (mean ± SD) and ranged from 0 % to 95 % (Table 5). Coverage varied substantially across content areas, with several key topics remaining poorly addressed (Table 2).

**Table 5:** Mean and range of readability, content, understandability, actionability, and quality across different characteristics.

	Year	Words	SMOG	Content	PEMAT-U	PEMAT-A	PEMAT total	modEQIP
All results (duplicates included)								
N	65	100	100	100	100	100	100	100
Mean	2022	4737	12.3	64 %	83 %	52 %	76 %	51 %
(SD)	(1.8)	(5982)	(1.5)	(26 %)	(10 %)	(39 %)	(15 %)	(25 %)
Range	2016–2024	40–24,253	10–17.9	0–95 %	62–94 %	0–100 %	42–95 %	0–86 %
Unique results								
N	23	47	47	47	47	47	47	47
Mean	2022	2581	12.6	49 %	80 %	37 %	70 %	40 %
(SD)	(2.0)	(4226)	(1.7)	(25 %)	(10 %)	(35 %)	(14 %)	(26)
Range	2016–2024	40–24,253	10–17.9	0–95 %	62–94 %	0–100 %	42–95 %	0–86 %
Query mean								
Breast reconstruction	2022	5797	11.7	72 %	86 %	65 %	81 %	59 %
(SD)	(2.3)	(6273)	(1.3)	(22 %)	(10 %)	(37 %)	(15 %)	(20 %)
DIEP flap	2022	3107	12.9	41 %	79 %	31 %	68 %	36 %
(SD)	(1.5)	(5901)	(1.5)	(24 %)	(10 %)	(36 %)	(14 %)	(26 %)
Mastectomy reconstruction	2022	4894	12.2	67 %	85 %	54 %	78 %	52 %
(SD)	(1.8)	(6023)	(1.3)	(26 %)	(8.6 %)	(40 %)	(15 %)	(25 %)
Breast reconstruction	2022	5988	12.3	67 %	83 %	56 %	77 %	57 %
(SD)	(1.8)	(7380)	(1.8)	(24 %)	(10 %)	(41 %)	(16 %)	(26 %)
Mastectomy breast reconstruction	2022	3902	12.3	69 %	84 %	51 %	76 %	53 %
(SD)	(1.7)	(3896)	(1.3)	(22 %)	(10 %)	(39 %)	(16 %)	(22 %)
Rank mean								
1–5	2021	5575	12.0	77 %	85 %	67 %	81 %	58 %
(SD)	(2.2)	(5203)	(0.9)	(20 %)	(9.7 %)	(34 %)	(14 %)	(19 %)
6–10	2022	5695	12.2	67 %	85 %	55 %	79 %	58 %
(SD)	(1.3)	(6770)	(1.5)	(24 %)	(9.7 %)	(42 %)	(16 %)	(22 %)
11–15	2022	5487	12.3	59 %	81 %	53 %	74 %	56 %
(SD)	(2.2)	(7537)	(1.8)	(29 %)	(12 %)	(38 %)	(18 %)	(26 %)
16–20	2023	2192	12.8	52 %	82 %	31 %	71 %	33 %
(SD)	(0.5)	(2982)	(1.6)	(23 %)	(8.3 %)	(36 %)	(13 %)	(25 %)
Author mean								
Academic (*N* = 20)	2021	1132	12.5	45 %	81 %	35 %	70 %	25 %
(SD)	(1.7)	(993)	(1.6)	(18 %)	(9.2 %)	(29 %)	(11 %)	(17 %)
Other (*N* = 27)	2022	3653	12.6	52 %	80 %	39 %	70 %	52 %
(SD)	(2.1)	(5297)	(1.7)	(29 %)	(11 %)	(39 %)	(16 %)	(25 %)
Target audience mean								
Patients (*N* = 43)	2022	2595	12.4	50 %	81 %	40 %	72 %	39 %
(SD)	(2.1)	(4363)	(1.6)	(24 %)	(9.4 %)	(34 %)	(13 %)	(24 %)
Other (*N* = 4)	2023	2369	14.8	40 %	64 %	0 %	50 %	55 %
(SD)	(0.0)	(1135)	(1.7)	(40 %)	(4.2 %)	(0 %)	(4.6 %)	(48 %)
Country mean								
USA (*N* = 44)	2022	2319	12.7	48 %	79 %	35 %	69 %	38 %
(SD)	(2.1)	(3944)	(1.6)	(25 %)	(10 %)	(34 %)	(14 %)	(25 %)
Canada (*N* = 1)	2023	1833	11.4	70 %	94 %	50 %	85 %	57 %
(SD)	(0.0)	(0)	(0.0)	(0 %)	(0 %)	(0 %)	(0 %)	(0 %)
United Kingdom (*N* = 1)	2023	14,788	10.0	90 %	94 %	100 %	95 %	79 %
(SD)	(0.0)	(0)	(0.0)	(0 %)	(0 %)	(0 %)	(0 %)	(0 %)
Australia (*N* = 1)	2023	2618	11.8	60 %	85 %	75 %	82 %	79 %
(SD)	(0.0)	(0)	(0.0)	(0 %)	(0 %)	(0 %)	(0 %)	(0 %)

Subgroup analysis revealed a significant difference in content scores across the five queries used in the study, χ² (4,*N* = 100) = 16.94, *p* = 0.002 (Table 6). Dunn’s test with Bonferroni correction indicated the content scores among ‘DIEP flap’ query results (41 % ± 24 %) were significantly lower than from all other queries (*p* < 0.05 in all four pairwise comparisons). No significant differences were found between the remaining query groups. The ‘breast reconstruction’ query had the highest content score (72 % ± 22 %) among results. Content coverage did not differ significantly between resources produced by academic authors and those produced by non-academic authors (*U* = 228, *p* = 0.365).

**Table 6:** Query Kruskal-Wallis tests and author type Mann-Whitney U tests across resource metrics.

	Rank	Words	SMOG	Content score	PEMAT-U	PEMAT-A	PEMAT total	EQIP
Query								
Chi-square		χ² = 9.629^a^	χ² = 7.366	χ² = 16.938^b^	χ² = 6.559	χ² = 8.285	χ² = 7.062	χ² = 9.104
Author								
U statistic	*U* = 657^b^	*U* = 138^b^	*U* = 255.5	*U* = 228	*U* = 257	*U* = 263	*U* = 268	*U* = 104^c^
*z*-score	*z* = −3.078^b^	*z* = −2.840^b^	*z* = −0.312	*z* = −0.906	*z* = −0.286	*z* = −0.158	*z* = −0.043	*z* = −3.640^c^

Kruskal–Wallis tests (χ²) were used for comparisons across search queries. Mann–Whitney U tests (U, z) were used for comparisons between academic and non-academic authors.

a *p* < 0.05.

b *p* < 0.01.

c *p* < 0.001.

Using Spearman’s *ρ*, significant relationships were observed between content scores and rank (*ρ* = −0.415, *p* < 0.001; i.e., higher content score was associated with higher search rank), word count (*ρ* = 0.795, *p* < 0.001), understandability scores (*ρ* = 0.566, *p* < 0.001), actionability scores (*ρ* = 0.440, *p* = 0.002), total PEMAT scores (*ρ* = 0.545, *p* < 0.001), and quality scores (*ρ* = 0.592, *p* < 0.001) (Table 7).

**Table 7:** Relationships between website measures of readability, content, understandability, actionability, and quality.

	Year	Words	SMOG	Content score	PEMAT-U	PEMAT-A	PEMAT total	EQIP
Rank	*ρ = 0.314*^a^	*ρ =* −*0.363*^b^	*ρ = 0.188*	*ρ =* −*0.415*^b^	*ρ =* −*0.259*^b^	*ρ =* −*0.361*^b^	*ρ =* −*0.302*^b^	*ρ =* −*0.325*^b^
Year		*ρ = 0.425*^a^	*ρ = 0.107*	*ρ = 0.249*	*ρ = 0.103*	*ρ = 0.317*	*ρ = 0.292*	*ρ = 0.260*
Words			*ρ =* −*0.115*	*ρ = 0.795*^b^	*ρ = 0.507*^b^	*ρ = 0.512*^b^	*ρ = 0.566*^b^	*ρ = 0.631*^b^
SMOG				*ρ =* −*0.222*	*ρ =* −*0.354*^a^	*ρ =* −*0.497*^b^	*ρ =* −*0.445*^b^	*ρ =* −*0.197*
Content score					*ρ = 0.566*^b^	*ρ = 0.440*^b^	*ρ = 0.545*^b^	*ρ = 0.592*^b^
PEMAT-U						*ρ = 0.509*^b^		*ρ = 0.168*
PEMAT-A								*ρ = 0.354*^a^
PEMAT total								*ρ = 0.284*

Spearman’s rho (*ρ*) is reported.

a *p* < 0.05.

b *p* < 0.01.

### Readability

The mean article length among unique resources was 2581 ± 4226 words and ranged from 40 to 24,253 words (Table 5). The mean SMOG reading level was 12.3 ± 1.7 and ranged from 10.0 to 17.9.

As shown in Table 6, Kruskal-Wallis testing indicated a significant difference in resource length across queries [χ² (4,*N* = 100) = 9.63, *p* = 0.047], with ‘breast reconstruction surgery’ having the longest results (5988 ± 7380 words) and ‘DIEP flap’ having the shortest (3107 ± 5901 words). However, Dunn’s test with a Bonferroni correction did not produce statistical significance across any of the pairwise differences. With respect to author type, resources written by non-academic authors were significantly longer (3653 ± 5297) than those written by academic authors (1132 ± 993) (*U* = 138, *p* = 0.005).

SMOG reading level did not vary significantly across queries [χ² (4,*N* = 100) = 7.37, *p* = 0.118] or between academic and non-academic authors (*U* = 256, *p* = 0.755) (Table 6).

Significant Spearman’s correlations were observed between resource length and rank (*ρ* = −0.363, *p* < 0.001), content coverage (*ρ* = 0.795, *p* < 0.001), understandability (*ρ* = 0.507, *p* < 0.001), actionability (*ρ* = 0.512, *p* < 0.001), total PEMAT (*ρ* = 0.566, *p* < 0.001), and quality (*ρ* = 0.631, *p* < 0.001). Similarly, SMOG reading level correlated significantly with understandability (*ρ* = −0.354, *p* = 0.015), actionability (*ρ* = −0.497, *p* < 0.001), and total PEMAT (*ρ* = −0.445, *p* = 0.002) (Table 7).

### Understandability and actionability

As detailed in Table 5, mean understandability, actionability, and total PEMAT scores were 80 % ± 10 %, 37 % ± 35 %, and 70 % ± 14 %, respectively.

Subgroup analysis (Table 6) revealed no significant differences in understandability [χ² (4,*N* = 100) = 6.56, *p* = 0.161], actionability [χ² (4,*N* = 100) = 8.29, *p* = 0.082], or total PEMAT scores [χ² (4,*N* = 100) = 7.06, *p* = 0.133] across queries. Similarly, there were no significant differences in understandability (*U* = 257, *p* = 0.775), actionability (*U* = 263, *p* = 0.875), or total PEMAT scores (*U* = 263, *p* = 0.966) between academic and non-academic authors.

Understandability and actionability were strongly correlated (*ρ* = 0.509, *p* < 0.001). There was also a significant Spearman’s correlation between understandability and rank (*ρ* = −0.259, *p* = 0.009), resource length (*ρ* = 0.507, *p* < 0.001), SMOG reading level (*ρ* = −0.354, *p* = 0.015), and content coverage (*ρ* = 0.566, *p* < 0.001). Actionability was significantly correlated with rank (*ρ* = −0.361, *p* < 0.001), resource length (*ρ* = 0.512, *p* < 0.001), SMOG reading level (*ρ* = −0.497, *p* < 0.001), content coverage (*ρ* = 0.440, *p* = 0.002), and quality (*ρ* = 0.354, *p* = 0.015). Finally, total PEMAT score was significantly correlated with rank (*ρ* = −0.302, *p* = 0.002), resource length (*ρ* = 0.566, *p* < 0.001), SMOG reading level (*ρ* = −0.445, *p* = 0.002), and content coverage (*ρ* = 0.545, *p* < 0.001). These relationships are shown in Table 7.

### Quality

The mean modEQIP score among unique resources was 40 % ± 26 % (Table 5). There was no significant difference in quality across queries on subgroup analysis [χ² (4,*N* = 100) = 9.10, *p* = 0.059] (Table 6). However, a Mann-Whitney U test revealed that the modEQIP score of resources authored by academic authors was significantly lower than that of resources authored by others (*U* = 104, *p* < 0.001).

Quality was significantly correlated with rank (*ρ* = −0.325, *p* < 0.001), resource length (*ρ* = 0.631, *p* < 0.001), content coverage (*ρ* = 0.592, *p* < 0.001), and actionability (*ρ* = 0.354, *p* = 0.015). It was not significantly correlated with SMOG reading level, understandability, or total PEMAT score (Table 7).

## Discussion

With nearly half of U.S. adults demonstrating limited health literacy and many patients seeking information online, the quality of digital patient education resources has meaningful implications for informed consent, satisfaction, and outcomes.^6^ Prior studies have raised concerns about the accuracy, readability, and comprehensiveness of online health information, yet few have examined these in the context of breast reconstruction using a multidimensional assessment framework.^10^^,^^12^ To our knowledge, this is the first study to comprehensively evaluate online breast reconstruction information across the five most commonly searched Google phrases. Although this analysis does not assess emerging large language models (LLMs), it establishes a benchmark for evaluating online patient education materials, as these emerging tools rely on the same underlying web-based content.

Broadly, we found moderate content coverage, with significant gaps in key topics. Readability was consistently poor, with average SMOG reading levels exceeding recommended thresholds. Understandability was high while actionability was lacking. Resource quality and transparency were also suboptimal. However, higher-ranked resources tended to score better across most metrics, suggesting some alignment between search engine visibility and overall quality.

### Content

Our analysis revealed substantial variability in content coverage across breast reconstruction resources, with essential topics being inconsistently addressed.^2^ Approximately half of topics identified as relevant were omitted on average. Autologous tissue and implant-based reconstruction, along with aesthetic outcomes, were covered in over 80 % of resources, reflecting their central relevance to patients considering the procedure.^2^ However, other key topics were often missed, with preoperative planning, adverse effects from neoadjuvant/adjuvant treatments, disease recurrence, fat grafting, and the use of acellular dermal matrices being discussed in fewer than 30 % of resources.^2^^,^^13^ This may be because authors feel these topics are less suitable for online presentation (and more suitable for individualized physician-patient discussion).

Notably, the content score for the ‘DIEP flap’ query was significantly lower than all other queries, with no significant differences observed among the remaining queries. This pattern suggests that as search terms become more specific—shifting from general reconstruction to particular reconstructive options—the corresponding resources tend to narrow their focus accordingly. In contrast, the shared terminology of other queries likely contributes to the consistently wider range of information pertinent to breast reconstruction on the whole when compared to more specific ‘DIEP flap’ resources.

Higher content scores were also associated with higher ranking search results. This may reflect greater alignment between content-rich resources and search engine optimization factors such as relevance, topical depth, and keyword usage.^17^ Content coverage was also strongly associated with resource length as addressing a broad range of topics naturally requires more writing. Similarly, content scores were positively correlated with understandability and actionability, likely highlighting how broad topic coverage provides better context to present practical guidance, enhancing both the clarity and applicability of information for patients. Finally, a positive correlation was observed between content scores and resource quality. Resources investing in broad coverage of relevant topics, may increasingly invest in other aspects of development, in turn promoting clearer authorship, disclosure practices, and patient-centered design. Interestingly, the lack of association between SMOG score and content coverage suggests comprehensive information may still be presented in accessible language through deliberate simplification of vocabulary and structure.^18^

### Readability

Results had an average reading grade level of 12.3, exceeding the National Institutes of Health (NIH) recommended 8th-grade reading level.^12^^,^^19^^,^^20^ The persistently poor readability seen across studies may reflect the inherent complexity of breast reconstruction, concerns of oversimplification, or perhaps more simply, lack of emphasis on readability during resource development.^21^^,^^22^ With readability being independent of content coverage, use of plain-language principles and readability guidelines, such as the Centers for Disease Control and Prevention Clear Communication Index, could help bridge the gap and improve clarity without compromising completeness.^23^

SMOG reading level was negatively correlated with both understandability and actionability scores, suggesting simplified language not only improves readability but also enhances instructional clarity and patient ability to act on information. Additionally, resource length was negatively correlated with rank, suggesting comprehensive and detailed resources are prioritized by search engines to satisfy user information needs and engagement metrics. Resource length was also positively correlated with content, understandability, actionability, and quality scores. These relationships suggest longer resources have more opportunity to address relevant topics, present clear and actionable guidance, while meeting transparency standards. Moreover, word count was the only metric associated with publication year, highlighting evolving standards in patient education and a shift toward providing increasingly comprehensive patient resources online.^24^ Interestingly, academic authors produced significantly shorter resources than non-academic authors, possibly indicating a preference for concise technical summaries over more lengthy explanations.

### Understandability and actionability

Across resources, understandability was consistently high. Each query achieved an average score above 70 %, used by previous studies as a threshold of adequate understandability.^11^^,^^18^ Understandability also remained robust across search ranks. Poor resource readability concomitant with high understandability reflects the separation between language complexity and presentation clarity, where integrating features such as headings and bullet points support patient navigation of the information.^7^ In contrast, actionability scores were globally low. A major contributor was the limited use of visual aids to illustrate actionable steps, as identified in previous studies.^10^^,^^11^

Both understandability and actionability demonstrated significant relationships with search rank, suggesting that Google’s ranking algorithm may prioritize clearer, actionable resources—or that user engagement reinforces their visibility.^25^ Interestingly, only actionability was correlated with quality scores, indicating that resources with greater emphasis on transparency and credibility may similarly prioritize providing patient-centric, actionable guidance. PEMAT scores did not differ significantly across queries, suggesting that features supporting understandability and actionability are consistent across both general and specialized queries.

### Quality

Quality scores were low across resources, indicating deficiencies in resource credibility and transparency. Particularly poor performance was noted in measures of financial disclosure (reported among 17 % of resources) and patient involvement in content creation (not reported at all).

Surprisingly, academic authors published resources with significantly lower quality scores versus non-academic authors. Academic institutions are typically held to high standards regarding financial disclosure and authorship transparency, particularly in formal publications. The lower transparency observed in academic-authored results may therefore reflect differences in institutional standards between academic and online publishing or a lack of translation from typical academic rigor to publicly accessible formats.

Quality scores were also significantly correlated with rank, with higher-quality resources ranked higher. This suggests that Google’s search algorithm rewards the publication trustworthiness signaled by high transparency and disclosure.

Together, these findings highlight a serious need for improved standards in the disclosure and transparency practices of online breast reconstruction resources, particularly for specialized content and academic-origin materials.

### Comparison to previous findings

Our findings align with and extend previous evaluations of online breast reconstruction resources.^12^^,^^13^ Marcasciano et al. (2019), using the expanded EQIP, similarly reported websites lacked key information on surgical risks and complications. Despite methodological differences we highlight persistent deficiencies in the comprehensiveness and quality of online patient education resources.

Poor readability is also seen in prior studies.^10^^,^^12^^,^^21^^,^^22^ Chen et al. reported elevated reading levels across online breast reconstruction resources. In a 20-year longitudinal study, Rooney et al., demonstrated a trend of consistently high readability levels in patient education materials across specialties, with minimal improvement over time. These studies underscore a longstanding, systemic issue in health communication, where patient resources continue to exceed recommended literacy standards, despite greater institutional focus on health literacy initiatives.

Finally, while Lynch et al. found no correlation between website quality and search rank, our study indicated significant positive relationships between rank and multiple quality metrics. This suggests potential improvements in how search engines prioritize higher-quality educational content, with top-ranked breast reconstruction resources exhibiting stronger content coverage, understandability, actionability, and transparency.

### Limitations

This study has limitations that may affect the generalizability of its findings. First, search results are dynamic, while our single-day data is only a temporal snapshot of online patient educational information. To assess the impact of search result turnover, we re-ran our search in September 2025 and verified the stability of results, with the majority of original results being present in this updated search. This suggests that the overarching conclusions of this study would have likely been the same had it been undertaken today. Second, the online information landscape has evolved with the rapid emergence of LLMs and AI-driven search platforms. While Google remains a common entry point for patients seeking health information and continues to shape initial exposure to resources, its Gemini-powered ‘AI Overviews’ are increasingly replacing manual review of search results for relevant information. Lastly, search results will vary for each patient’s query due to geographic targeting and personalization of algorithms based on trends, region, search engine, search history, and language.

Regarding metrics, the SMOG tool, while validated, may underestimate the complexity of medical terminology due to its reliance on syllable count. Binary content coverage scoring cannot capture insight regarding the extent of a subtopics discussion or comprehensively measure content quality. The EQIP tool’s validity was limited by our use of only its identification items, reducing comparability with studies using the full instrument.

## Conclusion

Online resources remain a key source of information for breast reconstruction patients, often turning to the internet as a supplemental source of education.^3^^,^^4^ This multimetric evaluation of online breast reconstruction resources revealed strong understandability and favorable search rankings, but significant weaknesses across readability, content coverage, actionability, and transparency. Despite high understandability, poor readability poses ongoing barriers to comprehension.^10,12,21,22^Inconsistent content, and omission of critical topics echo prior studies.^12^^,^^13^ The low actionability and limited transparency further undermine patient ability to use and trust online resources.^26^^,^^27^

Encouragingly, higher-quality resources tended to appear earlier in search rankings, and language complexity did not correlate with content breadth, indicating that comprehensive information can still be presented in plain language.^18^ Specific procedural queries performed worse across all domains, highlighting gaps in resource quality among niche patient searches. Improving online resources will require clearer language, broader content, and greater actionability, supported by clinicians who can reinforce key concepts and direct patients to reliable sources, particularly for specialized topics.^28^^,^^29^

## Ethical approval

This study did not require ethical approval.

## Funding

None.

## Declaration of competing interest

None.

## Appendix Supplementary materials

Image, application 1
