# Supplementary material for: Quality and accessibility of online patient self-education resources for breast reconstruction
Source: JPRAS Open. 2025 Dec 18;48:500–13. doi: 10.1016/j.jpra.2025.12.022 (PMC12811492; doi:10.1016/j.jpra.2025.12.022)
Supplement: Supplementary file 1 [file mmc1.docx]

**Supplemental Material**

**Example Query Results**

| <https://my.clevelandclinic.org/health/treatments/16809-breast-reconstruction> |
| --- |
| <https://www.plasticsurgery.org/reconstructive-procedures/breast-reconstruction> |
| <https://www.cancer.gov/types/breast/reconstruction-fact-sheet> |
| <https://www.cancer.org/cancer/types/breast-cancer/reconstruction-surgery.html> |
| <https://www.mayoclinic.org/tests-procedures/breast-reconstruction-flap/about/pac-20384937> |
| <https://www.yalemedicine.org/news/breast-reconstruction-after-cancer> |
| <https://www.komen.org/breast-cancer/treatment/type/surgery/breast-reconstruction/> |
| <https://www.ncbi.nlm.nih.gov/books/NBK470317/> |
| <https://www.webmd.com/breast-cancer/breast-reconstruction-after-mastectomy> |
| <https://www.brighamandwomens.org/surgery/plastic-surgery/reconstructive-procedures/breast-reconstruction> |
